# *Fragman*: an R package for fragment analysis

**DOI:** 10.1186/s12863-016-0365-6

**Published:** 2016-04-21

**Authors:** Giovanny Covarrubias-Pazaran, Luis Diaz-Garcia, Brandon Schlautman, Walter Salazar, Juan Zalapa

**Affiliations:** Department of Horticulture, University of Wisconsin, Madison, WI USA; Instituto Nacional de Investigaciones Forestales, Agricolas, y Pecuarias, Campo Experimental Pabellon, Aguascalientes, Mexico; USDA-ARS, Vegetable Crops Research Unit, University of Wisconsin, Madison, WI USA

**Keywords:** Fragment analysis, Genetic markers, R package, Least squares, Open source software

## Abstract

**Background:**

Determination of microsatellite lengths or other DNA fragment types is an important initial component of many genetic studies such as mutation detection, linkage and quantitative trait loci (QTL) mapping, genetic diversity, pedigree analysis, and detection of heterozygosity. A handful of commercial and freely available software programs exist for fragment analysis; however, most of them are platform dependent and lack high-throughput applicability.

**Results:**

We present the R package *Fragman* to serve as a freely available and platform independent resource for automatic scoring of DNA fragment lengths diversity panels and biparental populations. The program analyzes DNA fragment lengths generated in Applied Biosystems® (ABI) either manually or automatically by providing panels or bins. The package contains additional tools for converting the allele calls to GenAlEx, JoinMap® and OneMap software formats mainly used for genetic diversity and generating linkage maps in plant and animal populations. Easy plotting functions and multiplexing friendly capabilities are some of the strengths of this R package. Fragment analysis using a unique set of cranberry (*Vaccinium macrocarpon*) genotypes based on microsatellite markers is used to highlight the capabilities of *Fragman*.

**Conclusion:**

*Fragman* is a valuable new tool for genetic analysis. The package produces equivalent results to other popular software for fragment analysis while possessing unique advantages and the possibility of automation for high-throughput experiments by exploiting the power of R.

**Electronic supplementary material:**

The online version of this article (doi:10.1186/s12863-016-0365-6) contains supplementary material, which is available to authorized users.

## Background

Polymerase chain reaction (PCR)-based genetic markers such as microsatellite or simple sequence repeat (SSR), amplified fragment length polymorphism (AFLP), and single nucleotide polymorphism (SNP) markers have become essential in genetic research [1, 2]. Traditionally, fragment analysis has been the crucial first step for genetic research such as mutation detection, linkage and quantitative trait loci (QTL) mapping, genetic diversity and other genetic applications using molecular markers [2, 3]. During fragment analysis, fluorescently-labeled PCR amplified DNA fragments, which have been denatured by a chemical reagent (i.e., formamide), are injected along with an appropriate size standard into capillaries for electrophoresis size separation [4, 5]. Essentially, a high voltage charge applied to the PCR fragments promotes their migration along the capillaries from cathode to anode, where smaller fragments migrate faster than larger fragments [4]. When capillary electrophoresis is performed using ABI instrumentation (i.e., AB Genetic Analyzer, Applied Biosystems 3730), the size-separated PCR fragments are detected by reading their fluorescence intensity at different emission wavelengths and are recorded as FSA (.fsa) files, which can be used in downstream analyses [5]. FSA files are typically loaded into specialized licensed and platform dependent software which compare the capillary electrophoresis position and fluorescent intensity of the sample PCR fragments and the size standard to estimate the number of nucleotides in the sample. Here, we present a new freely available and platform independent R package, *Fragman*, for performing fragment analysis. *Fragman* accurately and automatically scores DNA fragment lengths in diversity panels and biparental populations and transforms the observed lengths into formats required for further genetic analysis in other software such as GenAlEx, JoinMap and OneMap [6–8]. *Fragman* was compared with other fragment analysis software such as GeneMarker®, and we obtained similar genotyping results, but with superior automation and throughput scoring capabilities.

## Implementation

The workflow of the program consists in the use of 5 basic steps: 1) Reading the data using the function *storing.inds*, which loads the FSA files and smooth the data; 2) matching the ladder with the function *ladder.info.attach*, which finds the correct peaks in the size-standard channel corresponding to the expected DNA sizes to fit a linear model in order to calibrate the samples and attaches such information to the R environment for subsequent use; 3) creating panels with the function *overview2*, which is used to generate bins of alleles by marker; 4) scoring peaks and assigning DNA sizes with the function *score.easy*, which locates the peaks provided in the panel and assigns the size in base pairs for each sample; and 5) Exporting to different formats with the functions *get.scores* and *jm.conv*.

In the special case where samples cannot be correctly matched by the automatic *ladder.info.attach* function because the samples are too noisy, the *ladder.corrector* function has been provided to allow the users to manually correct noisy samples. Also, an extra function named *overview,* allows the users to manually score the samples through regular functions available by default in R, such as the *locator* function.

### Reading the files using the storing.inds function

This function is a wrapper function of *read.abif* from the *seqinr* package, which reads FSA files [9]. In addition, *storing.inds* extracts the channel information containing the fluorescent intensities from the DNA capillary electrophoresis up to any number of colors. Fourier frequency transformation (FFT) techniques are applied for smoothing the data in order to enhance the signal, pull up adjustments are performed to diminish channel to channel noise, and peak correction for saturated peaks over 8000 relative fluorescent units (RFUs) is performed as other licensed software do [10].

### Matching the ladder with peaks found using the ladder.info.attach function

The user must supply a numeric vector containing the expected base pairs sizes of the ladder fragments co-migrating with the sample DNA fragments during capillary electrophoresis. The program calculates the first derivative of the intensity vector for the channel of fluorescence containing the size standard, and finds the point where the slope approximates zero (i.e., $\frac{dy}{dx}=0$: where *y* is the intensity with respect to the index position *x*) using the *rle* function from the *base* package [11]. An iterative procedure using least squares creates parallel models and model with the highest correlation is then selected. This procedure confidently finds the correct fluorescent peaks in all the FSA files to match them with the expected DNA sizes of the size standard, and finally uses a linear model of the form y = Xβ + ε to assign a base pair value to each index of the intensity vector where y is the response defined as the expected DNA sizes for the ladder, X is the incidence matrix for fixed effects, β is the vector of fixed effects for the polynomial regression until the fifth order to account for the migration differential between DNA pieces of different sizes [12].

An example of how to create a vector with the expected sizes and size the samples is:

### Panel generation by using the overview2 function

After matching the fragments in the size standard to their expected lengths, all sizing information must be loaded into the R environment. Subsequently, the best way to score samples is by creating panels across the capillary regions in the channels where the PCR products of interest were read [10]. Peaks can be easily visualized by using the *overview2* function, which generates a plot overlapping all the fluorescent signals for all loaded FSA files in order to manually select peaks for creating panels of allele bins.

The plot allows graphical assessment of zero slope peaks (i.e., alleles) present in the set of selected FSA files (i.e., individuals of a population). This plot can be used to create a panel of allele bins that are passed to the *score.easy* function by manually clicking on the desired zero slope peaks and obtaining the potential DNA sizes in the population using the *locator* function installed by default in R package *base* [11]. This function can create the panel as:

### Scoring peaks and assigning DNA sizes with score.easy function

The core of the program relies on this function. The function uses information from the FSA files read by *storing.inds* and the size standard calibration information generated by *ladder.info.attach* to perform a zero slope peak search in the channels/fluorescent colors specified by the user and assigns size in base pairs for such peaks. In addition, a panel of zero slope peaks (i.e., alleles) supplied by the *overview2* function reduces the search to a subset of potential DNA fragment sizes.

The user can implement this function as follow:

In this line of code the user provides the original data read in step 1 (“my.data”), chooses to score the first channel (cols = 1), provide the panel created in step 3 (“my.panel”), and includes the ladder specified in step 2 (“my.ladder”).

### Exporting to different formats with the functions get.scores and jm.conv

One of the strengths of the *Fragman* package is the capability to convert to other formats commonly used in genetic analysis such as JoinMap®, OneMap and GenAlEx. The implementation of such function is straight forward after the scoring step. To extract the results for a marker scored in data frame format as any other licensed software we can use: where “newdata” is a user-friendly presentation of the data in a column format. The second line of code shows how the data in a column format is easily converted to JoinMap® format.

## Results and discussion

To determine the accuracy of the software, we compared the analyses of 1000 raw FSA from four different cranberry populations (3 biparental mapping populations and 1 genetic diversity panel) in *Fragman* and the commercial software GeneMarker®.

### Loading and cleaning of the data

**Fig. 1:**
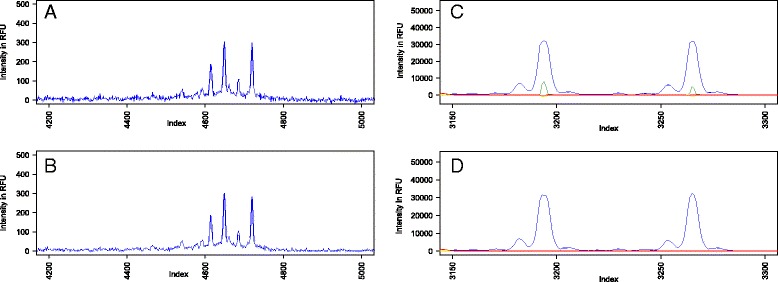
Effect of Fourier transformation on smoothing and pull up correction during fragment data analysis using *Fragman*. **a** Raw data extracted from the FSA file and (**b**) data treated after Fourier transformation. Raw peak prior to (**c**) and after (**d**) pull up correction to decrease noise between channels

Initially, we started a project by loading the data into R using the function *storing.inds* [9]. The function extracted fluorescent intensity information from all channels/fluorescent colors creating a data frame that was smoothed by applying a Fourier transform using only the top 40 % of lowest frequencies (Fig. 1). A pull up correction was then applied to each channel to decrease channel to channel noise (Fig. 1). First, all channels were added to identify capillary regions containing zero slope peaks. Then, a window was set across the capillary regions containing zero slope peaks to identify the channel where each peak had the largest intensity and then subtract the noise caused by such peaks in other channels.

### Testing the correlation of the ladder sizing

**Fig. 2:**
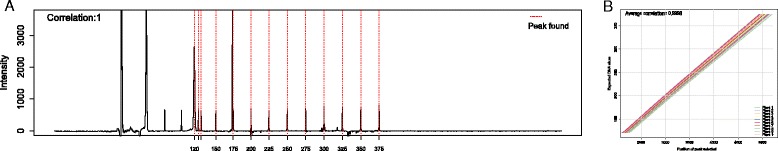
Size standard calibration features of *Fragman*. In (**a**) An example of zero slope peaks selected in the 4^th^ (r*ed*) channel, showing a correlation greater than 0.9999 with the expected size standard fragment lengths selecting the right peaks. In (**b**) correlation between expected DNA fragment in the Rox375 size standard (y-axis) and observed zero slope peaks for the size standard selected by *Fragman* in 10 random FSA files (x-axis)

**Fig. 3:**
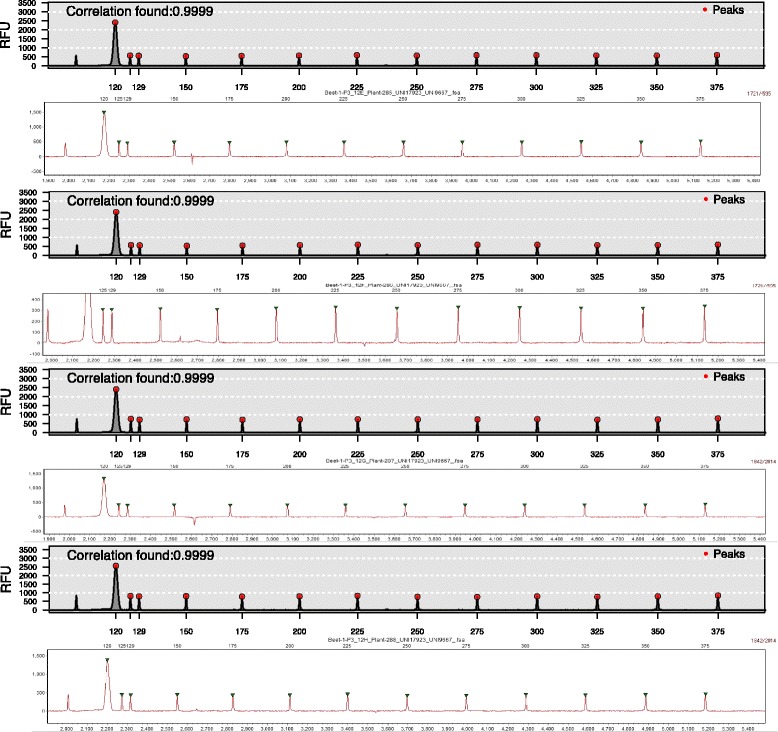
Ladder sizing comparison. Comparison between the size standard calibration capabilities of the *Fragman* package (odd positions to the bottom) and the licensed GeneMarker® software (even positions to the bottom) for the same 4 samples. In x axis the base pair size is displayed whereas the intensity is plotted in the y axis. Samples scored with dotted red lines correspond to *Fragman* whereas samples scored with green dots correspond to GeneMarker®

Size standard calibration in *Fragman* in the channel containing the size standard relies on detection of all possible combinations of zero slope peaks that surpass an initial fluorescent threshold. Subsequently, the program conducts an iterative procedure and selects the combination with the highest correlation with expected size standard fragment sizes. In order to assess the accuracy of size standard zero slope peak selection and sizing in *Fragman*, two different size standards were tested, ROX375 (GeneScan™) and LIZ500 (GeneScan™), and size standard calibration was performed in a diversity panel using 24 microsatellite markers in 2 channels (1000 FSA files total) using FAM (blue) and HEX (green) fluorescent dyes. Size standard calibration performance in *Fragman* was compared to GeneMarker®. First, we validated the *Fragman* size standard calibration function*, ladder.info.attach*, by extracting the correlation observed between the expected size standard fragment lengths and the selected size standard zero slope peaks determined for 1000 FSA files. We found that they yielded an average correlation of 0.99951, indicating that the expected fragment lengths and those observed using *storing.inds* matched (Fig. 2). Using both ROX375 (GeneScan™) and LIZ500 (GeneScan™), we found a perfect match between the size standard zero slope peaks and their predicted fragment length in base pairs when compared with commercial software GeneMarker® sizing [10] (Fig. 3). Similar to commercial software, *Fragman* had problems detecting the correct combination of ladder peaks when the ladder’s relative fluorescent units (RFUs) was lower than 150, which dramatically increased the number of peaks in the channel containing the size standard due to noise, making it impossible to compute such a large number of zero slope peak combinations. To deal with low fluorescent signals in the channel containing the size standard, *Fragman* uses an exhaustive sampling strategy of 15,000 random peaks in the size standard channel to find the best combination of possible size standard zero slope peaks. In our tests, this strategy resulted in a correct solution about 94 % of the time.

### Panel validation

**Fig. 4:**
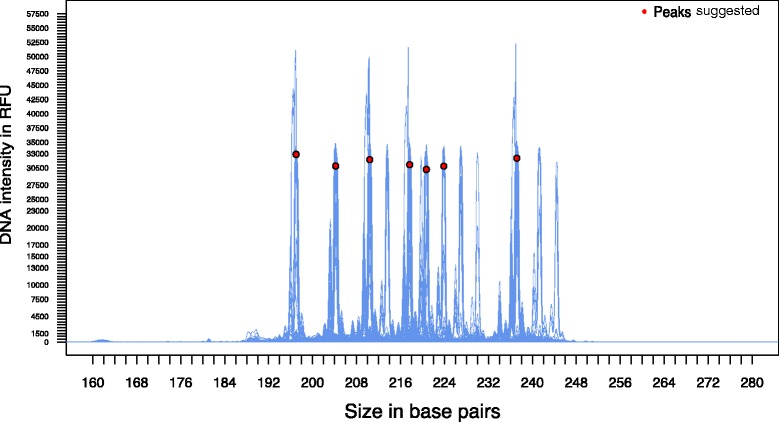
Panel construction in *Fragman* package. Visual output of the *overview2* function implemented in *Fragman* to create panels of potential alleles found in a population. The function overlaps the fluorescent intensities in all the loaded FSA files for the selected channel. Here, the fluorescent emission intensity of FAM labeled DNA fragments in 288 diploid plants from a diversity panel are plotted to show the allelic variability for this genetic marker. The function by default suggests and returns all peaks with minor allele frequency (MAF) > 0.05 along with a plot which can be used interactively with the use of the *locator* function to manually select alleles

Identical DNA zero slope peaks (i.e., alleles), even though exactly the same fragment length, do not necessarily occur at the exact same base pair index in two different FSA files due to differential migration of fragments during capillary electrophoresis. This problem can lead to different allele calls between FSA files when in reality they are the same allele. Therefore, most commercial software such as GeneMarker® and GeneMapper® have an option for creating allele panels with scoring windows that account for differential migration in order to make size fragment scoring faster and more accurate. For example, creating a panel for an allele of 200 bp in length with a zero slope index of 200.3 bp and a window of 0.5 bp will allow every peak in that range to have the same allele call. We implemented a similar approach for creating panels of allele bins with in the *overview2* function by overlapping the curves of our samples to determine the appropriate window size for each allele bin. This approach allows *Fragman* to correct for differential migration and to perform genotypic calls more efficiently (Fig. 4). The *locator* function from the R *base* package is implemented in *overview2* to allow the user to click on the desired zero slope peaks (alleles) for allele bin creation. This was an effective means to extract a vector of user-supplied alleles to create panels to be used in *score.easy* and also making panel construction much simpler and faster than other methods implemented in the licensed fragment analysis software.

### Scoring and multiplexing

**Fig. 5:**
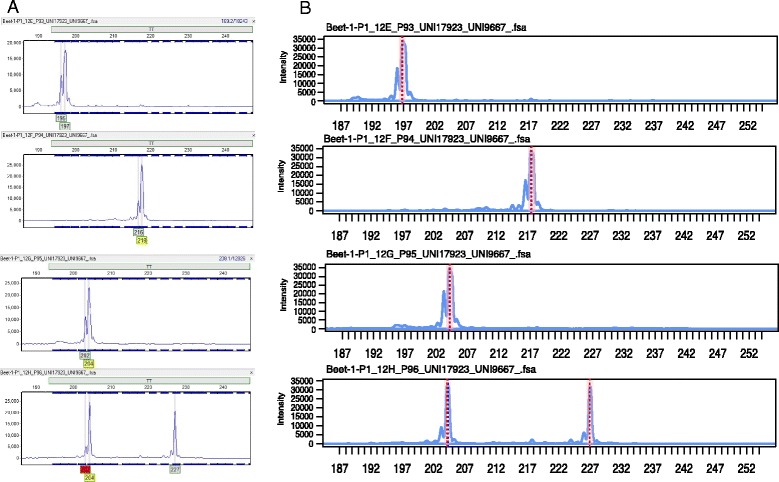
Scoring comparison between *Fragman* and GeneMarker®. **a** Example of GeneMarker® scoring compared to (**b**) *Fragman* scoring (zero slope peak selection) in four random FSA files, showing the ability of *Fragman* to better differentiate between real and noisy peaks compared with GeneMarker®, which usually picks noisy peaks as real

Incorporation of multiple fluorescence dyes into DNA during PCR amplification using M13 allows researchers to reduce the cost of fragment analysis by pooling PCR products from multiple markers into the same capillary, and then later separating the fragments by marker into multiple channels based on their fluorescence at unique emission wavelengths [13]. Furthermore, multiple markers can be combined into a single channel as long as there is no overlap in the allele ranges of the designed panels. We tested the ability of the *Fragman* package to deal with any number of dyes and markers per channel compared to commercial software used for fragment analysis. When using the *score.easy* function in *Fragman* and GeneMarker® to determine the fragment lengths of several markers in 1000 individual FSA files derived from 3 biparental populations and a diversity population, we found that up 98 % of the samples were scored correctly using *Fragman*, which implements the functions *left.cond* and *right.cond*, whereas only 85 % where accurately scored using the commercial software GeneMarker® prior to manually deleting or adjusting miscalled peaks (Fig. 5).

*Fragman* analysis software was designed to select the best alleles based on the ploidy of the organism; however, this process tends to be highly error-prone due to differential patterns of PCR amplification that lead to background peaks and stuttering resulting from incomplete 3' nucleotide addition [5]. For polyploid organisms, *Fragman* will call all zero slope peaks above a selected threshold when users are scoring non-diploid organisms. Additional functions were added to *Fragman* for formatting the fragment lengths measured in *score.easy* according to the required inputs for the common linkage mapping and diversity analysis software, JoinMap® [6], OneMap [8] and GenAlEx [7]. These functions allow users to avoid time-consuming manual conversion of data types between software, and the same functions can also be used to convert SNP calls from by genotyping by sequencing (GBS) to JoinMap® and OneMap as well [14].

### Comparing Fragman with other existing fragment analysis software

*Fragman* offers a full fragment analysis pipeline comparable to GeneMarker® consisting of three main steps: 1) FSA files are read and sized according to a ladder, 2) data is scored using bins established by the user, and 3) data is exported as an excel file to be used for linkage mapping or other genetic analyses. To our knowledge, there is no other free software package available that performs all the same services as *Fragman*. The only other freely available, platform independent software comparable to *Fragman* is Peak Studio, written in Java, but during our tests it often crashed with large sample numbers and it did not allow for automation and is no longer being updated [15]. Other R packages that could be comparable to *Fragman* include Genomatic (unpublished), Biostrings (unpublished), and MsatAllele [16], but they all rely on licensed software such as GeneMapper® or GeneMarker® or the windows dependent STRAND [17] (Additional file 1). Thus, these packages independently do not provide a full fragment analysis pipeline, which includes sizing, peak calling, and data export to other software for genetic analyses. *Fragman* is a complete fragment analysis, software package, which provides most if not all of the services available in licensed software while also implementing several new previously unavailable and automated functions useful in downstream genetic analyses.

## Conclusions

We have developed an R package with the ability to perform efficient and accurate fragment analysis by taking advantage of the power of R [11], which provides extra graphical and high-throughput capabilities for high dimensional projects.

Major features of *Fragman* are:

1. The ability to directly load FSA files for fragment analysis
2. The ability to work with any DNA standard (ladder)
3. The ability to automatically and simultaneously analyze multiple samples
4. Flexibility in the scoring preference (i.e. manual or automatic scoring)
5. Accurate automatic scoring of diversity and biparental populations
6. Additional tools for analyzing and converting the data into other formats

## Availability and requirements

Project name: *Fragman*: an R package for fragment analysis

Project home page: https://cran.r-project.org and http://cggl.horticulture.wisc.edu

Operating system(s): Platform independent

Programming language: R

Other requirements: R > 2.0

License: GPL-3

### Ethics (and consent to participate)

Not applicable.

### Consent to publish

Not applicable.

## Additional file

Additional file 1: Features of other computer software available to analyze fragment data. (DOCX 126 kb)

## Abbreviations

*Bp*: base pair
*DNA*: Deoxyribonucleic acid
FAM, HEX, NED, ROX and PET: dyes used for DNA sequencing. All four dyes can be excited at a single wavelength (488 nm), but emit at distinctly different wavelengths
*FSA*: File associated with Applied Biosystems, usually output of DNA fragment analysis. A type of FASTA file format
MAF: minor allele frequency
*M13*: universal primer for multiplexing
*SNP*: single nucleotide polymorphisms
*SSR*: simple sequence repeat
