# Supplementary material for: Fragman: an R package for fragment analysis
Source: BMC Genet. 2016 Apr 21;17:62. doi: 10.1186/s12863-016-0365-6 (PMC4839125; doi:10.1186/s12863-016-0365-6)
Supplement: Additional file 1: — Features of other computer software available to analyze fragment data. (DOCX 126 kb) [file 12863_2016_365_MOESM1_ESM.docx]

**Additional File 1**

Other available open source software can be found on the internet presumably performing fragment analysis. In this file we review briefly why they were not used in this particular research and provide links to access them in case are useful for readership analysis. Bugs in the source code, platform dependency, low high throughput applicability, and attribution of actions not implemented were the main reasons for developing this open source software and compare it with the most famous licensed software GeneMarker®.

We have resumed the fragment analysis pipeline and four steps and clarify why other open-source software available in the web usually confounded as fragment analysis software is not or was not feasible to use. This brief analysis, hopefully will provide readership with a clue of when or when not to use a specific software.

The Fragment analysis pipeline can be summarized in the next steps:

1. DNA samples sequenced in ABI machinery.
2. Resulting FSA files are read in a scoring software and sized according to a ladder
3. Data is scored using bins established by the user.
4. Data is exported as an excel file to be used for evolution, mapping or other genetic analysis.

Available free software on the web related to fragment analysis:

**A) Genomatic**

Genomatic documentation can be found on the website:

<http://people.oregonstate.edu/~knausb/software/Genomatic_users_manual_v03.pdf>

This program *doesn’t read the FSA itself (steps 1 and 2)* but is a software to organize data previously scored in a licensed ABI genotyping software and make graphs of it. The manual mentions explicitly; “*We score our data using ABI's Genotyper, but you can probably use other software as long as you can get it into our format”*. In summary it also depends from licensed software to do the first part of the fragment analysis but can perform step 3 and 4 of the pipeline.

In addition, the package is not published and is not maintained by the CRAN (The official repository of R packages). No sample data is included in the package.

**B.1) MsatAllele**

MsatAllele is a computer package built on R to visualize and bin the raw microsatellite allele size distributions. It does perform step 3 and 4 from the pipeline but *depends on the program STRAND for performing first steps*.

MsatAllele was published in Journal of Heredity 2009:100(3):394–397. The program is no longer maintained in CRAN but still can be downloaded at:

<https://cran.r-project.org/web/packages/MsatAllele/index.html>

**B.2) STRAND**

STRand is software developed and used at the University of California, Davis' Veterinary Genetics Lab. It analysis DNA fragment length polymorphism samples run on fluorescence based gels and capillaries. Is a GUI. It performs steps 1 and 2 from the pipeline but it is platform dependent (*only Windows-based*), and was not designed for high throughput analysis. The program can be found at:

<https://www.vgl.ucdavis.edu/informatics/strand.php>

**C) RFLPtools**

RFLPtools provides functions to analyse DNA fragment samples. It was designed to *perform analysis on gel images*. It uses information previously scored to create graphs and infer differences among individuals based on alleles differences. *It does not read FSA files from ABI machinery, it does not size samples according to ladder and does not score based on customized bins.*

It depends on licensed software for initial steps. I cite documentation from the package; “…it analyses data generated with software package Gene Profiler 4.05 (Scanalytics Inc.))”

<http://cran.fhcrc.org/web/packages/RFLPtools/RFLPtools.pdf>

The software was published in Molecular Ecology Resources (2013) 13, 726–733

<http://onlinelibrary.wiley.com/doi/10.1111/1755-0998.12094/epdf>

Therefore, we consider Fragman to be a unique in his kind among freely available software and because of that was only comparable to commercial software such as GeneMapper® or GeneMarker®.
